# Stationary *Lactococcus cremoris*: Energetic State, Protein Synthesis Without Nitrogen and Their Effect on Survival

**DOI:** 10.3389/fmicb.2021.794316

**Published:** 2021-12-17

**Authors:** Sieze Douwenga, Rinke J. van Tatenhove-Pel, Emile Zwering, Herwig Bachmann

**Affiliations:** ^1^TiFN, Wageningen, Netherlands; ^2^Systems Biology Lab, Amsterdam Institute for Molecules, Medicines and Systems, Vrije Universiteit Amsterdam, Amsterdam, Netherlands; ^3^Department of Biotechnology, Delft University of Technology, Delft, Netherlands; ^4^NIZO, Ede, Netherlands

**Keywords:** stationary phase, single cell, survival, protein synthesis, proton gradient, energetic state, starvation

## Abstract

During storage and ripening of fermented foods, *Lactococcus cremoris* is predominantly in a non-growing state. *L. cremoris* can become stationary due to starvation or acidification, and its metabolism in these non-growing states affects the fermented product. Available studies on the response of *L. cremoris* to acid and starvation stress are based on population level data. We here characterized the energetic state and the protein synthesis capacity of stationary *L. cremoris* cultures at the single cell level. We show that glucose starved stationary cells are energy-depleted, while acid-induced stationary cells are energized and can maintain a pH gradient over their membrane. In the absence of glucose and arginine, a small pH gradient can still be maintained. Subpopulations of stationary cells can synthesize protein without a nitrogen source, and the subpopulation size decreases with increasing stationary phase length. Protein synthesis capacity during starvation only benefits culturability after 6 days. These results highlight significant differences between glucose starved stationary and acid-induced stationary cells. Furthermore, they show that the physiology of stationary phase *L. cremoris* cells is multi-facetted and heterogeneous, and the presence of an energy source during stationary phase impacts the cells capacity to adapt to their environment.

## Introduction

*Lactococcus cremoris* [formerly *Lactococcus lactis* subsp. *cremoris* (Li et al., 2021)] is well-known for its use in food fermentations like cheese and yogurt (Kelleher et al., 2017; Pereira et al., 2020). During storage and ripening of these fermented products, *L. cremoris* spends weeks to months in a non-growing state. The cellular metabolism in this non-growing state can have a big impact on product properties, and therefore there is great interest in understanding the physiology in non-growing and stationary states of *L. cremoris* (van de Guchte et al., 2002; Papadimitriou et al., 2016; Nugroho et al., 2020).

**FIGURE 1:**
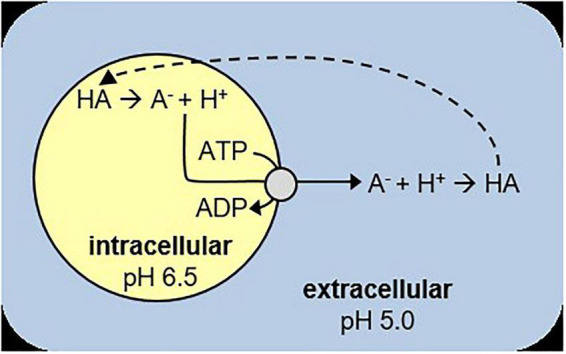
Schematic overview of weak-acid uncoupling. HA is the protonated acid, A^–^ the deprotonated acid. The dashed line indicates passive diffusion of HA into the cell. A^–^ and H^+^ are actively exported, which requires ATP.

In a typical batch culture *L. cremoris* metabolizes sugars to lactic acid, thereby decreasing the pH until the sugars are depleted (glucose starved stationary) (Supplementary Information Section 1; Supplementary Figure 1). When sugars are present in excess, cells will become stationary due to lactic acid stress (acid-induced stationary) (Rallu et al., 2000). At pH 6.6 and lower, both growing and acid-induced stationary *L. cremoris* cells maintain a pH gradient over their membrane (Even et al., 2002). Their intracellular pH does decrease with the extracellular pH as more lactic acid is produced, due to a process called weak-acid uncoupling. In this process the undissociated lactic acid form (pK_*a*_ = 3.9) passively diffuses into the cell, where it dissociates into the membrane impermeable lactate^–^ and H^+^. The cell in turn must export lactate^–^ and H^+^ to maintain a high intracellular pH, usually with the help of an H^+^-ATPase (Hutkins and Nannen, 1993; Šušković et al., 2010). Once outside, lactate^–^ and H^+^ will form the undissociated lactic acid form again, resulting in a futile cycle (Hutkins and Nannen, 1993; Cotter and Hill, 2003; Figure 1). This process eventually causes lactic acid related growth arrest, as the decrease in intracellular pH and increase in intracellular lactate concentrations reduce the glycolytic enzyme activity, while weak acid stress increases the maintenance energy requirements (Kashket, 1987; Nannen and Hutkins, 1991; Even et al., 2002; Carvalho et al., 2013).

Over the years, several studies aimed to better understand the physiology of acid-induced and glucose starved stationary *L. cremoris*. Next to exporting protons, glucose starved stationary cells synthesize stress-related proteins to deal with the increased protein and DNA damage (e.g., chaperones, proteases, and DNA repair proteins) (Papadimitriou et al., 2016). However, if growth has stopped due to acidification, these proteins are no longer produced (Budin-Verneuil et al., 2005). Two important lactococcal species are *L. lactis* and *L. cremoris*. One distinguishing feature of *L. lactis* over *L. cremoris* is the ability to catabolize arginine to ornithine and ammonia through the ADI pathway (Wels et al., 2019). This process yields 1 ATP per arginine which can be used for H^+^ translocation. Furthermore, the production of ammonia directly increases the intracellular pH by binding H^+^ (Cunin et al., 1986; Budin-Verneuil et al., 2003). Lactococcal taxonomy is somewhat confusing due to a disparity that arises between genotype and phenotype-based descriptions. This leads to strains that are of the genotype *L. cremoris* but which have an *L. lactis* phenotype. One of the best described lactococcal strains, MG1363, is genotypically a *L. cremoris* while it has an *L. lactis* phenotype. The *lactis* phenotype of this strain can be of relevance to stationary cells, as arginine utilization in this phenotype influences ATP production and the intracellular pH.

Catabolization of arginine to produce ATP in glucose starved cells, is known to increase the viability (Stuart et al., 1999; Brandsma et al., 2012). How cells exactly use the ATP to prevent cell death is not clear, as other studies report that the availability of energy rich compounds like ATP and PEP alone did not influence viability during starvation (Otto et al., 1985; Poolman et al., 1987). The increased survival of glucose starved cells in the presence of arginine is probably also not related to the capacity to synthesize protein, at least for the first few days of starvation (Thomas and Batt, 1969; Hartke et al., 1994).

While these studies give a good idea about the population response to acid and starvation stress, it was shown in recent years that microbial stress responses can be heterogeneous within a clonal population (Han and Burgess, 2010; Gasch et al., 2017; Zhang et al., 2020). This is of interest for a better mechanistic understanding of the stress response itself, but it also potentially impacts applications such as food fermentations and the use of *L. cremoris* as a cell factory.

We here characterized the energetic state and the protein synthesis capacity of stationary *L. cremoris* MG1363 cultures at the single cell level. Our results show that glucose starved stationary cells are energy-depleted, while acid-induced stationary cells are energized. In both, glucose starved stationary and acid-induced stationary cultures, a subpopulation of cells is able to synthesize protein even in the absence of a nitrogen source. During starvation, this protein synthesis capacity only benefits culturability after 6 days.

## Materials and Methods

### Strains and Media

*Lactococcus cremoris* MG1363 (Wegmann et al., 2007) and *L. cremoris* MG1363_GFP (van Tatenhove-Pel et al., 2019), the latter of which contains a genomically integrated GFP that is constitutively expressed, were used throughout this study. While MG1363 is genotypically a *cremoris* strain it does have a *L. lactis* phenotype and is able to utilize arginine (Wels et al., 2019). Strains were grown in Chemically Defined Medium (CDM) described by Price et al. (2019), supplemented with 0.09 w/v% glucose (stationary due to starvation) or with 0.45 w/v% glucose (stationary due to acidification). For plate counting, CDM supplemented with 0.09 w/v% glucose and 1 w/v% agarose was used. Cultures and plates were incubated at 30°C, without shaking.

### Flow Cytometry Measurements

GFP-signals of single cells were measured using an Accuri C6 flow cytometer (BD Biosciences, San Jose, CA, United States), after excitation at 488 nm and measuring emission with a 533/30 nm optical filter. A standard sample volume was measured that typically resulted in the collection of 10 000 to 220 000 events, and for which sample means of the GFP signals were calculated.

To analyze whether cells could maintain a pH gradient, we centrifuged samples for 2 min at a relative centrifugal force (rcf) of 16,000 × *g* and resuspended the pellets in phosphate buffered saline (PBS) of pH 7.5 or pH 4.7. The PBS was supplemented with glucose (0.5 w/v%) and/or lactate (20, 35 or 70 mM) and/or chloramphenicol (30 mg/L) when required. For data-normalization the fluorescence signal at pH 7.5 was taken as the maximal value, based on a previously published calibration curve (van Tatenhove-Pel et al., 2019).

### Analytical Procedures and Statistical Analysis

Optical density was measured at 600 nm (OD_600_). We used colorimetric glucose tests (MQuant^®^, Merck KGaA, Darmstadt, Germany) to determine if glucose was exhausted. When applicable sample size, standard deviation (SD) or standard error of the mean (SEM) are indicated.

### Acidification and Growth at Six Arginine Concentrations From 1.4 to 8.3 mM

To determine acidification of the medium we supplemented CDM with 1:1,000 of 0.4 w/v% 5(6)-Carboxyfluorescein BioReagent (Sigma-Aldrich) suspended in 10 v/v% ethanol in demineralized water (final 5(6)-Carboxyfluorescein concentration of 0.0004 w/v%) (Bachmann et al., 2012). Carboxyfluorescein fluorescence positively correlates with the pH.

After overnight pre-culture in CDM with 5 mM glucose, cells were diluted 1:1,000 in fresh CDM with 5 mM glucose supplemented with one of the following arginine concentrations: 1.4 mM (the standard arginine concentration in CDM), 2.8, 4.2, 5.5, 6.9, or 8.3 mM. The inoculated media were transferred to a black 384 wells plate (200 μL/well) with a transparent bottom (Greiner Bio-One). The plate was placed in a plate-reader (Tecan Safire II) at 30°C without shaking, where fluorescence (excitation: 485/20 nm; emission: 520/20 nm) and optical density at a wavelength of 600 nm (OD_600_) were measured every 6 min for 30 h.

### Kill-Curve With Translation Inhibitor

Kill curves were obtained by incubating glucose starved stationary *L. cremoris* MG1363 cultures in CDM without carbon source, with and without erythromycin (30 mg/L) and chloramphenicol (30 mg/L) at pH 6.5 and pH 5.4. Cultures were kept at 30°C for 16 days, with periodic sampling for colony forming units (CFU) determination. The antibiotic stability was checked by periodically sampling each tube, and adding glucose to a concentration of 0.45 w/v% to this sample. If no growth occurred while CFU determinations indicated that viable cells were present, this indicated that the antibiotics were still active.

## Results

### Glucose Starved Stationary Cells Do Not Maintain a pH Gradient Over the Membrane

To assess the effect of lactic acid production by *L. cremoris* on its intracellular pH, we used a Green Fluorescent Protein (GFP) based method. *L. cremoris* MG1363_GFP contains a single genomically integrated GFP gene, that is constitutively expressed (van Tatenhove-Pel et al., 2019). Only the deprotonated form of GFP is fluorescent (Kneen et al., 1998; Han and Burgess, 2010). The equilibration of the intracellular and extracellular pH with the membrane-uncouplers valinomycin and nigericin allows the calibration of the fluorescence signal of *L. cremoris* MG1363_GFP (Supplementary Information Section 2; van Tatenhove-Pel et al., 2019). On the basis of such a calibration curve (Supplementary Figure 2), we estimated the intracellular pH of stationary *L. cremoris* MG1363_GFP. For this, cells were grown with initial glucose concentrations ranging from 0.09 to 0.55 w/v%, resulting in increasing stationary phase lactate concentrations.

**FIGURE 2:**
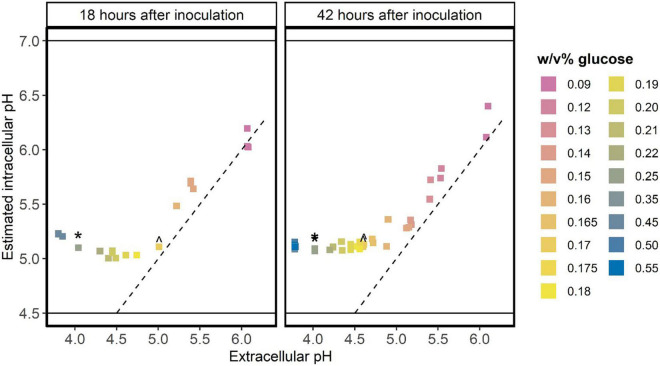
Glucose starved stationary cells do not maintain a pH gradient over the membrane. *L. cremoris* MG1363_GFP was grown in CDM with glucose concentrations ranging from 0.09 w/v% to 0.55 w/v% (indicated by colors), leading to differences in the final pH of the stationary cultures. At two time-points in stationary phase [18 h (*n* ≥ 1) and 42 h (*n* = 2) after inoculation] the pH of the cultures and the fluorescence of the cells was measured. As the initial glucose concentration might affect the GFP content of cells, we normalized the GFP signal of stationary cells using their GFP signal at pH 7.5 (maximal fluorescence signal of cells). This normalized fluorescence of cells was used to estimate the intracellular pH, following a calibration curve (Supplementary Information Section 2). This plot shows the measured extracellular pH versus the estimated intracellular pH. The dashed line indicates where the intracellular pH equals the extracellular pH. Solid horizontal lines indicate the regime wherein we can accurately estimate the intracellular pH (Supplementary Information Section 2). ^ initial glucose concentration of 0.17 w/v%. * initial glucose concentration of 0.25 w/v%.

In batch cultures with initial glucose concentrations up to 0.17 w/v%, the estimated intracellular pH was similar to the extracellular pH when cells go into stationary phase (Figure 2). This indicates that glucose starved stationary cells cannot maintain a pH gradient, which might be caused by a lack of energy. At initial glucose concentration between 0.17 and 0.35 w/v%, the estimated intracellular pH of stationary cells is higher than the measured extracellular pH, although we did not detect residual glucose in cultures initiated with 0.17–0.25 w/v% glucose. When the initial glucose concentration was higher than 0.35 w/v%, the intracellular pH increases compared to the intracellular pH values at initial glucose concentrations between 0.17 and 0.35 w/v%. This indicates that these cells can maintain a pH gradient and have energy, which might be due to the presence of glucose in these acid-induced stationary cultures.

### Glucose Starved Stationary Cells Consume Arginine

**FIGURE 3:**
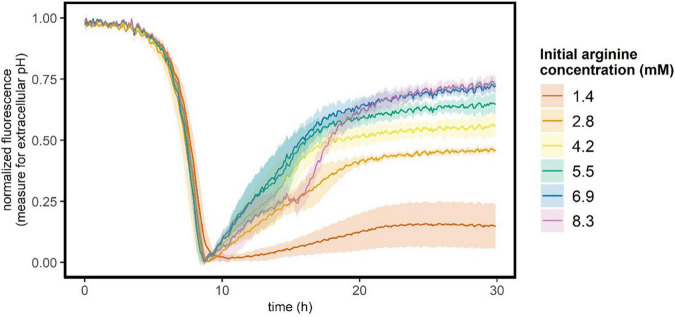
Extracellular pH of growing and glucose starved stationary *L. cremoris* cultures. *L. cremoris* was grown in CDM with six initial arginine concentrations (see legend), and the carboxyfluorescein signal (proxy for the extracellular pH) was followed in time. In the standard CDM 1.4 mM arginine is present. Lines show the mean, and shades de sd (*n* = 28 for 1.4 mM arginine, *n* = 4 for all other arginine concentrations). Corresponding OD_600_ values are shown in Supplementary Figure 3.

In the medium that we used, arginine is present in excess (1.4 mM) and can be used to generate ATP and ammonia, which increases the intracellular pH. To verify that this was not the cause of the observed pH gradient, we investigated the role of arginine on glucose starved stationary cultures. When arginine is consumed to produce ATP, ammonia is released. As ammonia is a base, arginine consumption will increase the extracellular pH, which can be measured as an increase in the fluorescence signal of the carboxyfluorescein that was added to the medium. Hence, to measure arginine consumption we followed the extracellular pH of glucose starved stationary cultures with six initial arginine concentrations ranging from 1.4 to 8.3 mM (in the standard medium 1.4 mM arginine is present). In the first 9 h, *L. cremoris* cells grew and acidified the medium (Figure 3). After 9 h glucose is depleted, cells stop growing (Supplementary Figure 3) and the extracellular pH increases again. This pH increase is larger and more rapid when the initial arginine concentration is higher, suggesting that when glucose is depleted cells metabolize arginine to generate ATP and ammonia. However, after roughly 20 h the extracellular pH became constant again, suggesting arginine depletion. This is sooner than we measured the pH gradient in Figure 2 right panel, hence cells can maintain a pH gradient both in the absence of glucose and arginine.

### Early-Stationary Glucose Starved Cells Are Still Energized

We hypothesized that longer periods of starvation would result in lower pools of energy rich molecules (e.g., glucose, ATP, and/or PEP). To study this, we determined the capacity of glucose starved stationary cells to maintain their intracellular pH in the presence of weak acid uncoupling in PBS at pH 4.7. Under these conditions the intracellular pH of cells will drop and the fluorescence of cells will decrease, unless they actively maintain a pH gradient, which requires energy (van Tatenhove-Pel et al., 2019). We varied the starvation period (growing, early- and late-stationary) by varying the inoculation density [OD_600_ of 10^–5^, 10^–4^, 10^–3^, 10^–2^, and 10^–1^ Arbitrary Units (AU)] of cultures that were inoculated at the same time and measured after identical incubation times. This resulted in starvation periods ranging from 0 to 15 h.

**FIGURE 4:**
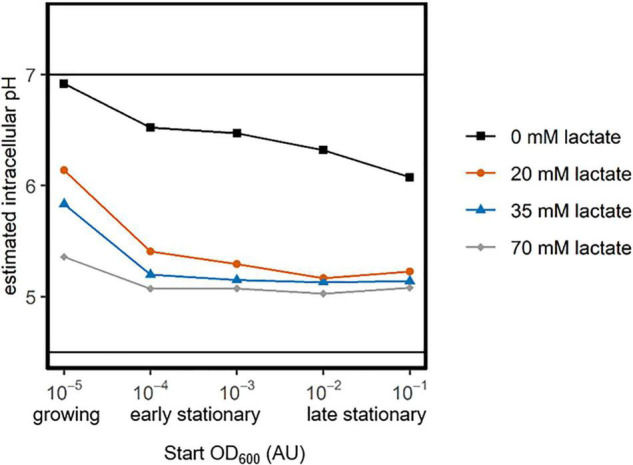
Maintenance of a membrane pH-gradient by growing, early- and late-stationary cells. *L. cremoris* MG1363_GFP was inoculated at five OD_600_ values (*x*-axis). After overnight growth, cells that were inoculated at an OD_600_ of 10^–5^ were still growing, while the other cultures were early- or late-stationary, due to starvation (categories below *x*-axis). Cells were washed and transferred to PBS of pH 7.5 (as a control) and to PBS of pH 4.7 with four lactate concentrations (color legend). The fluorescence of cells was measured directly after acid exposure. The fluorescence in PBS of pH 4.7 was normalized based on the fluorescence at pH 7.5. We used a calibration curve and this normalized fluorescence to estimate the intracellular pH, which is shown on the *y*-axis. Solid horizontal lines indicate the regime wherein we can accurately estimate the intracellular pH (Supplementary Information Section 2).

As the pH was set to 4.7 for all measurements the results show that it is the increasing lactic acid concentration that reduces the intracellular pH of the cells (Figure 4). This indicates that weak acid uncoupling (rather than just a low pH) is the largest contributor to intracellular acidification. Furthermore, the intracellular pH of exponential and early-stationary cultures is higher than that of late-stationary cells, indicating that exponential and early-stationary cells have more capacity to maintain a pH gradient over their membrane when exposed to weak-acid stress.

### Availability of Glucose Allows Cells to Maintain a Membrane pH Gradient

**FIGURE 5:**
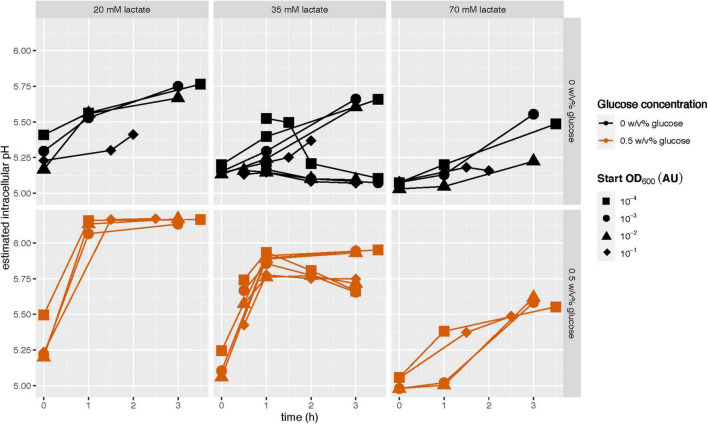
The ability of early- and late-stationary cells to maintain a pH gradient in the presence and the absence of glucose. Glucose starved stationary *L. cremoris* MG1363_GFP were washed and transferred to PBS of pH 7.5 (as a control) with and without glucose, and to PBS of pH 4.7 with and without glucose (color legend) and with three lactate concentrations (panels). The 35 mM experiments were done in duplicate, the experiments with the other two lactate concentrations were done once. The fluorescence of cells was measured in time (*x*-axis). The fluorescence in PBS at pH 4.7 was normalized based on the fluorescence at pH 7.5. We used a calibration curve and this normalized fluorescence to estimate the intracellular pH, which is shown on the *y*-axis. The fluorescence can be accurately measured between pH 4.5 and 7 (Supplementary Information Section 2).

To verify that the observed decrease in intracellular pH with increasing starvation duration was caused by a cell’s depleted energy reserves (Figure 4), we hypothesized that the addition of glucose should allow cells to maintain a larger pH gradient, even after they had been stationary for 15 h. To test this, we added glucose to cells suspended in PBS with lactic acid, and followed their intracellular pH in time. In the absence of glucose some cultures could increase their intracellular pH, but this increase was small and inconsistent (Figure 5). However, in the presence of glucose the cultures could indeed increase their intracellular pH. When we looked into the distributions underlying the averages of Figure 5, we observed a homogeneous response. Their final intracellular pH and the rate of increase does depend on the lactic acid concentration, but not on their stationary-phase length (four inoculation concentrations ranging from OD_600_ 10^–4^ to 10^–1^ AU). Next to glucose, also arginine can be used by cells to build-up and maintain a pH gradient (Supplementary Table 1).

### Stationary Cells Only Require a Carbon Source to Synthesize Protein

**FIGURE 6:**
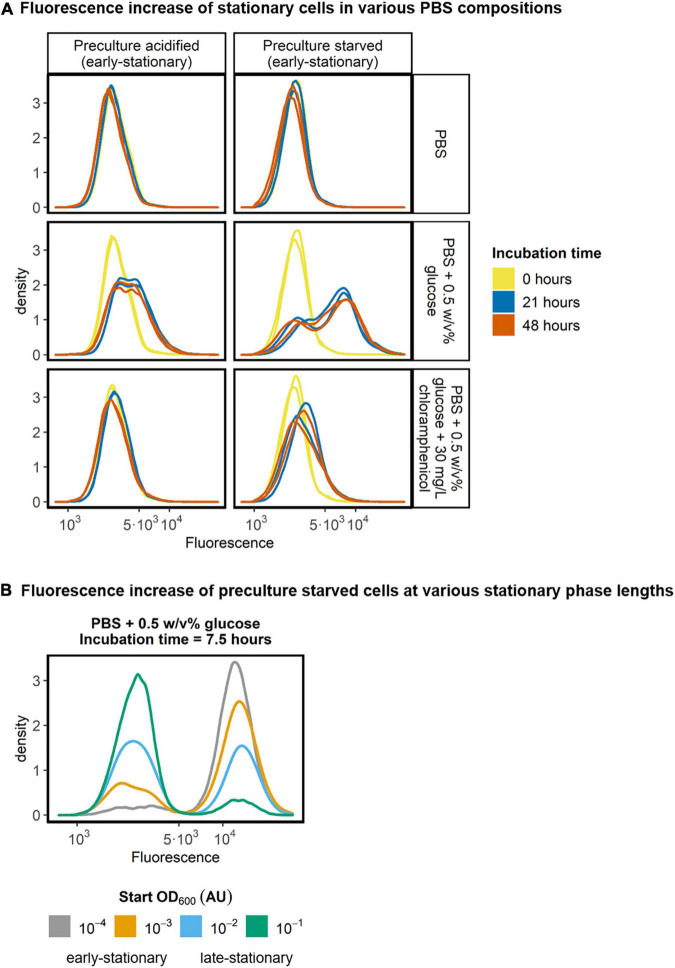
Fluorescence of stationary *L. cremoris* cells in PBS. **(A)** Acid-induced stationary and glucose starved stationary pre-cultures of *L. cremoris* MG1363_GFP (early-stationary) were diluted to 10^7^ cells/mL in PBS, PBS + 0.5 w/v% glucose and PBS + 0.5 w/v% glucose + 30 mg/L chloramphenicol. The pH of the PBS was 7.5 in all these conditions, to ensure a high intracellular pH in all cells throughout the incubation and measurement. The fluorescence of the cells was measured after 0, 21, and 48 h of incubation in PBS, and the resulting density plots (*n* = 2, with on average 100,000 cells measured per sample) are shown. **(B)** To analyze the effect of stationary phase length on the increase in the fluorescence of cells, glucose starved pre-cultures with four stationary phase lengths, classified as early- or late-stationary, were incubated for 7.5 h in PBS with 0.5 w/v% glucose and their fluorescence was measured. Resulting density plots (*n* = 1, with on average 55,000 cells measured per sample) are shown.

To test whether stationary *L. cremoris* retains protein synthesis capacity, we incubated glucose starved early-stationary and acid-induced early-stationary cells that constitutively expressed GFP in PBS of pH 7.5. We followed their fluorescence in time using flow cytometry. The cells were incubated in PBS of pH 7.5 and fluorescence was determined at three time points. We found that without any nutrients in PBS, the fluorescence signal of *L. cremoris* remained constant (Figure 6A, top row). However, when PBS was supplemented with glucose, a subpopulation of cells increased their fluorescence signal, indicating an increase of their GFP content (Figure 6A, middle row). Degradation of GFP could in theory weaken the observed fluorescence increase. However, GFP is a relatively stable protein, meaning that we detected most of the GFP produced, regardless of the time between GFP synthesis and measurement (Ormö et al., 1996; Mateus and Avery, 2000). To confirm the increase in the fluorescence signal originates from protein synthesis rather than other mechanisms, e.g., the maturation of a fluorescent protein, we did control experiments where we inhibited protein translation by adding the translation inhibitor chloramphenicol. The results show that the fluorescence increase was not observed in the presence of the translation inhibitor, corroborating that the increase is indeed caused by the production of new GFP by the early-stationary cells (Figure 6A, bottom row). Interestingly, acid-induced early-stationary cells produce less protein than glucose starved early-stationary cells. The supply of nutrients through cell lysis is not expected to play a major role in these cultures, as the cell concentration in PBS was low (10^7^ cells/mL), and constant in time. As the PBS used did not contain a nitrogen source these results indicate that in the presence of glucose only, stationary *L. cremoris* MG1363_GFP cells make new protein.

The protein synthesis in stationary cells was not homogeneous in the population, but two subpopulations were observed (Figure 6A, middle row). To see if the size of the subpopulation was affected by the length of the stationary phase, we measured the fluorescence of early- and late-stationary glucose starved cells after incubation in PBS with glucose for 7.5 h. The results show that the shorter the cells were in stationary phase, the larger the subpopulation of cells that can produce GFP (Figure 6B and Supplementary Figure 5). The strength of the fluorescence signal of the subpopulation of protein producing cells appears to be the same when comparing 7.5 h incubation time (Figure 6B) with 21 and 48 h incubation time of preculture starved cells (Figure 6A, middle row, right column). This suggests that when cells can produce protein, they will always produce roughly the same amount and do so before 7.5 h, regardless of their time in starvation. Interestingly, when glucose starved cells were transferred to fresh medium without an available carbon source, they also produced GFP, in amounts similar to those in PBS supplemented with glucose (Supplementary Figure 4).

### Protein Synthesis Capacity Only Affects Survival After More Than 7 Days of Starvation

**FIGURE 7:**
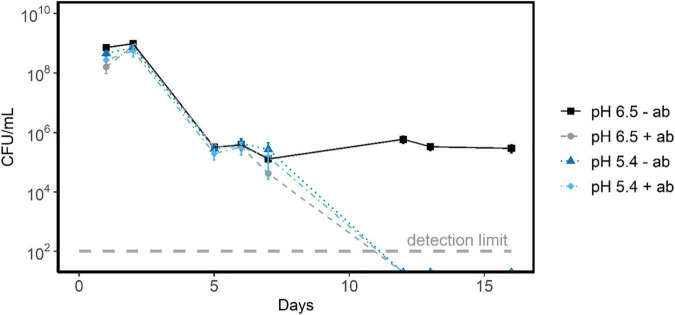
Survival of glucose starved stationary L. *cremoris* cells in the presence and absence of translation inhibitors. This plot shows the CFU/mL versus starvation time in days for glucose starved stationary L. *cremoris* MG1363 cells in CDM with translation inhibitors erythromycin and chloramphenicol (+ ab) at pH 5.4 (light blue) and pH 6.5 (gray). As a control cells were starved without translation inhibitors (−ab), also at pH 6.5 (black) and pH 5.4 (dark blue). Errors bars show SEM (*n* = 3), the detection limit of the assay was 10^2^ cells/mL.

To identify whether the observed protein synthesis capacity in stationary phase affected the survival during starvation, we tested the effect of translation inhibitors on survival during starvation at pH 6.5 and at pH 5.4 (set with lactic acid). For this we prepared acid-induced stationary *L. cremoris* MG1363 cells, and transferred them to medium without a carbon source, either with or without translation inhibition. The decrease in CFU over the first 6 days was similar in all conditions tested (pH 5.4, pH 6.5; with and without antibiotic) (Figure 7). In all conditions the CFU decreases by roughly 3 log scales (99.9%). Hence, for the first 6 days, we conclude that while protein synthesis can occur (Figure 6), this did not convey a benefit to cells resuming growth. On day 7, cells exposed to pH 6.5 with translation inhibitor show a slightly higher death-rate than cells without translation inhibition. After 12 days, glucose starved stationary cells at pH 5.4 were unable to resume growth, regardless of antibiotic being present. Interestingly, from 7 to 16 days, glucose starved stationary cells at pH 6.5 without translation inhibitors showed a stable viability. In contrast, glucose starved stationary cells at pH 6.5 with translation inhibitors were unable to resume growth, indicating that protein synthesis conveys a benefit to survival at pH 6.5 when starvation lasts more than 6 days. At pH 4.5, glucose starved stationary cells were not able to resume growth irrespective if they were incubated with or without a translation inhibitor.

## Discussion

During storage and ripening of fermented products, cells spend weeks to months in stationary phase and their metabolism in this non-growing state affects the product properties. To better understand this physiological state, we analyzed the energy level and protein synthesis capacity of glucose starved stationary and acid-induced stationary *L. cremoris* MG1363 cells at the single cell level.

One open question pertains to the activity of glycolysis in stationary cells. There is currently no consensus in the literature whether the glycolytic enzyme activity remains constant or decreases in starved stationary *L. lactis* cells. Kunji et al. reported a 65% reduction in glycolytic enzyme activity of galactose starved *L. cremoris* after 8 h. This was only restored in the absence of the translation inhibitor, chloramphenicol, upon galactose addition (Kunji et al., 1993). Redon et al. found the opposite for glucose starved *L. lactis* after 3.5 h, showing full retention of glycolytic enzyme activity (Redon et al., 2005). In this study we determined for *L. cremoris* whether it retains glycolytic enzyme activity up to 15 h of starvation or after being acid-induced stationary for up to 36 h.

In contrast to Kunji et al. our results show that when glucose starved stationary *L. cremoris* cells are incubated in PBS supplemented with lactate and glucose, they retain the capacity to rebuild and maintain a proton gradient over their membrane for at least 15 h (Figure 5; Kunji et al., 1993). Similarly, acid-induced stationary cells maintained a proton gradient over their membrane when glucose was present, even after being in stationary phase for ∼36 h (Figure 2). This is consistent with the work of Even et al., who showed that acid-induced stationary cells maintained a pH gradient over their membrane for more than 55 h (Even et al., 2002). Together, these findings suggest that both acid-induced and glucose starved stationary cells retain the capacity to actively metabolize glucose at sufficiently high rates to maintain a proton gradient over their membrane for more than 15 h.

In the absence of glucose, cells have to rely on previously produced energy rich molecules. Previous studies report that glucose starved stationary *L. cremoris* populations were fully depleted of such energy rich compounds within 0.5 to 2 h (Otto et al., 1985; Poolman et al., 1987). We here show that the entire population of glucose starved early stationary cells retains the ability to maintain a proton gradient when exposed to lactic acid stress (20 mM lactic acid at pH 4.7, Figure 4). Late stationary cells (8–13 h of starvation) could no longer maintain a pH gradient, which is longer than previously reported. Potentially, different strains or medium compositions result in different energy depletion times. Additionally, starved *L. cremoris* cells gradually use up residual arginine in the medium for ATP production (Figure 3; Redon et al., 2005). Aside from generating ATP, arginine catabolism is known to increase the intracellular pH (Hutkins and Nannen, 1993; Cotter and Hill, 2003). Hence, a gradual arginine depletion might explain the gradual decline in the ability of *L. cremoris* cells to maintain a pH gradient over their membrane.

When we measured the intracellular and extracellular pH of both glucose starved stationary and acid-induced stationary cells, we observed three regimes (Figure 2). In the first one (initial glucose concentration higher than 0.25 w/v%) cells were acid-induced stationary, and they could maintain a pH gradient. In the second regime (initial glucose concentration lower than 0.17 w/v%) cells were glucose starved stationary, and they could not maintain a pH gradient. Between these two regimes (initial glucose concentration 0.17 – 0.25 w/v%) cells were glucose starved stationary, but they could still maintain a pH gradient. Highly similar combinations of intracellular and extracellular pH values were reported previously, for cells that had been glucose starved stationary up to 79 h (Even et al., 2002). Our results show that the capacity to maintain a pH gradient between initial glucose concentrations of 0.17 and 0.25 w/v% is not linked to the presence of arginine. We conclude this because arginine is depleted 20 h after glucose depletion, but a pH gradient was still maintained 30 h after glucose depletion.

At the population level glucose starved stationary *L. cremoris* ML3 cells are reported to show limited protein synthesis in phosphate buffer supplemented with only glucose or arginine (Thomas and Batt, 1969). We here used constitutively expressed GFP as an indicator for protein synthesis on the single cell level. With this we studied the protein synthesis capacity of starved and acid-induced stationary cells, by transferring these cells to PBS with and without glucose and PBS with glucose with and without chloramphenicol. We detected hardly any fluorescence increase in translationally blocked cells (chloramphenicol), showing that protein synthesis was the main driver of fluorescence increase in the other conditions tested. However, we found that a subpopulation of glucose starved stationary cells can produce protein when we provide them with glucose in the absence of amino acids (Figure 6). The fraction of cells capable of producing protein drops rapidly with the time cells have been stationary (Figure 6B).

For the protein producing subpopulation, all synthesis appears to occur within the first 7.5 h. Potentially, a subpopulation of cells might have a limited pool of intracellular amino acids available, either in the form of free amino acids or through proteins being degraded. However, compared to our results in PBS we do see that GFP increases by roughly the same amount in fresh medium without available carbon source. Despite the fact that all amino acids are available in this medium, the data shows also here that GFP increases only in a subpopulation (Supplementary Figure 4). Interestingly, we never observed this GFP increase in glucose starved stationary cells kept in the glucose depleted growth medium (in contrast to PBS). This might be due to the gradually declining energy state of the cells as arginine is consumed to depletion during starvation (Figure 3; Redon et al., 2005). The observed bimodal distribution in protein synthesis capacity may be caused by different mechanisms. For instance, genes in the arginine deaminase pathway are described to be heterogeneously expressed (Redon et al., 2005; Han and Burgess, 2010). Next to the role of arginine, depletion of another compound in spent medium may explain the lack of protein synthesis in spent medium. Protein synthesis capacity in acid-induced stationary cells also shows heterogeneity, but plateaus at a lower level than in glucose starved stationary cells (Figure 6A, left column).

One could hypothesize that the cells that do not produce protein are simply dead, and that the fraction of dead cells increases with the stationary phase length. However, all cells were able to maintain a pH gradient when exposed to PBS of pH 4.7 supplemented with lactate and glucose. This indicates that also cells that were not able to synthesize new protein had an intact membrane and were metabolically active. It is therefore tempting to speculate that the subpopulation without protein synthesis capacity may be linked to cellular dormancy states, such as persister formation (cells can be revived) or the viable but non-culturable state (cells cannot be revived anymore), which have been reported for *L. cremoris* MG1363 (Redon et al., 2005; Ganesan et al., 2007; Han and Burgess, 2010; van Tatenhove-Pel et al., 2019). However, such links would need to be investigated with dedicated experiments.

The bimodal distribution of protein synthesis capacity in these cultures argues against a gradual depletion of protein synthesis capacity in single cells with increasing starvation duration. To verify whether this ability to synthesize protein conveyed a survival advantage for starving *L. cremoris* cells, we transferred acid-induced stationary cells to starvation medium with translation inhibitor (cells were not able to adapt to starvation prior to translation inhibition). Protein synthesis capacity only benefited starvation survival at pH 6.5 after 6 days. For glucose starved stationary cells at pH 5.4, we did not find a beneficial effect of protein synthesis. However, we cannot exclude that differences in survival may have been present between day 7 and 12. Our findings agree with the work of Hartke et al., who showed a highly increased death-rate of translationally blocked cells starting after 10 days of starvation (Hartke et al., 1994). They also state that blocking translation after 3 h of starvation has a lower negative effect on starvation survival. This is consistent with our work, considering that our culture was already stationary due to glucose starvation prior to translation inhibition, potentially allowing it to synthesize some of the proteins required for survival over the first 6 days. Kunji et al. showed that protein synthesis occurs for the first 1–2 h of starvation, after which this declines rapidly (Kunji et al., 1993). Similarly, we found that the fraction of cells capable of synthesizing protein in PBS in the presence of glucose declines rapidly with stationary phase length.

The physiology of stationary phase *L. cremoris* cells is multi-facetted and heterogeneous. Major determinants for cellular behavior are whether cells reached stationary phase due to starvation or due to acidification. The fact that we find distinct differences at the single cell level adds to the complexity. Improving industrial production processes that are affected by the physiology of stationary phase cells can take these factors into account, and relatively small changes in medium composition could have large effects on the stationary phase physiology of *L. cremoris*.

## Data Availability Statement

The original contributions presented in the study are included in the article/Supplementary Material, further inquiries can be directed to the corresponding author.

## Author Contributions

SD, RTP, and HB conceived the study, designed the experiments, interpreted the data, and wrote the manuscript. SD, RTP, and EZ carried out the laboratory experiments. All authors contributed to the article and approved the submitted version.

## Conflict of Interest

This project was partially organized by and executed under the auspices of TiFN, a public-private partnership on precompetitive research in food and nutrition. Funding for this research was obtained from FrieslandCampina (Wageningen, Netherlands), CSK Food Enrichment (Wageningen, Netherlands), and the Top-sector Agri&Food. HB is employed by NIZO a contract research organization. The remaining authors declare that the research was conducted in the absence of any commercial or financial relationships that could be construed as a potential conflict of interest.

## Publisher’s Note

All claims expressed in this article are solely those of the authors and do not necessarily represent those of their affiliated organizations, or those of the publisher, the editors and the reviewers. Any product that may be evaluated in this article, or claim that may be made by its manufacturer, is not guaranteed or endorsed by the publisher.

## References

[B1] Bachmann H. Fischlechner M. Rabbers I. Barfa N. Dos Santos F. B. Molenaar D. (2013). Availability of public goods shapes the evolution of competing metabolic strategies. *Proc. Natl. Acad. Sci. U.S.A.* 110, 14302–14307. 10.1073/pnas.1308523110 23940318 PMC3761572

[B2] Brandsma J. B. van de Kraats I. Abee T. Zwietering M. H. Meijer W. C. (2012). Arginine metabolism in sugar deprived *Lactococcus lactis* enhances survival and cellular activity, while supporting flavour production. *Food Microbiol.* 29 27–32. 10.1016/j.fm.2011.08.012 22029915

[B3] Budin-Verneuil A. Maguin E. Auffray Y. Ehrlich S. D. Pichereau V. (2003). An essential role for arginine catabolism in the acid tolerance of *Lactococcus lactis* MG1363. *Lait* 84 61–68.

[B4] Budin-Verneuil A. Pichereau V. Auffray Y. Ehrlich D. Maguin E. (2005). Proteomic characterization of the acid tolerance response in *Lactococcus lactis* MG1363. *Proteomics* 5 4794–4807. 10.1002/pmic.200401327 16237734

[B5] Carvalho A. L. Turner D. L. Fonseca L. L. Solopova A. Catarino T. Kuipers O. P. (2013). Metabolic and transcriptional analysis of acid stress in *Lactococcus lactis*, with a focus on the kinetics of lactic acid pools. *PLoS One* 8:e68470. 10.1371/journal.pone.0068470 23844205 PMC3700934

[B6] Cotter P. D. Hill C. (2003). Surviving the acid test: responses of gram-positive bacteria to low pH. *Microbiol. Mol. Biol. Rev.* 67 429–453. 10.1128/MMBR.67.3.429-453.2003 12966143 PMC193868

[B7] Cunin R. Glansdorff N. Piérard A. Stalon V. (1986). Biosynthesis and metabolism of arginine in bacteria. *Microbiol. Rev.* 50 314–352. 10.1128/mmbr.50.3.314-352.1986 3534538 PMC373073

[B8] Even S. Lindley N. D. Loubière P. Cocaign-Bousquet M. (2002). Dynamic response of catabolic pathways to autoacidification in *Lactococcus lactis*: transcript profiling and stability in relation to metabolic and energetic constraints. *Mol. Microbiol.* 45 1143–1152. 10.1046/j.1365-2958.2002.03086.x 12180931

[B9] Ganesan B. Stuart M. R. Weimer B. C. (2007). Carbohydrate starvation causes a metabolically active but nonculturable state in *Lactococcus lactis*. *Appl. Environ. Microbiol.* 73 2498–2512. 10.1128/AEM.01832-06 17293521 PMC1855592

[B10] Gasch A. P. Yu F. B. Hose J. Escalante L. E. Place M. Bacher R. (2017). Single-cell RNA sequencing reveals intrinsic and extrinsic regulatory heterogeneity in yeast responding to stress. *PLoS Biol.* 15:e2004050. 10.1371/journal.pbio.2004050 29240790 PMC5746276

[B11] Han J. Burgess K. (2010). Fluorescent indicators for intracellular pH. *Chem. Rev.* 110 2709–2728. 19831417 10.1021/cr900249z

[B12] Hartke A. Bouche S. Gansel X. Boutibonnes P. Auffray Y. (1994). Starvation-induced stress resistance in *Lactococcus lactis* subsp. *lactis IL*1403. *Appl. Environ. Microbiol.* 60 3474–3478. 10.1128/aem.60.9.3474-3478.1994 16349399 PMC201836

[B13] Hutkins R. W. Nannen N. L. (1993). pH Homeostasis in Lactic Acid Bacteria1. *J. Dairy Sci.* 76 2354–2365.

[B14] Kashket E. R. (1987). Bioenergetics of lactic acid bacteria: cytoplasmic pH and osmotolerance. *FEMS Microbiol. Rev.* 3 233–244.

[B15] Kelleher P. Bottacini F. Mahony J. Kilcawley K. N. Sinderen Dv. (2017). Comparative and functional genomics of the *Lactococcus lactis* taxon; insights into evolution and niche adaptation. *BMC Genomics* 18:267. 10.1186/s12864-017-3650-5 28356072 PMC5372332

[B16] Kneen M. Farinas J. Li Y. Verkman A. (1998). Green fluorescent protein as a noninvasive intracellular pH Indicator. *Biophys. J.* 74 1591–1599. 10.1016/S0006-3495(98)77870-1 9512054 PMC1299504

[B17] Kunji E. R. S. Ubbink T. Matin A. Poolman B. Konings W. N. (1993). Physiological responses of *Lactococcus lactis* ML3 to alternating conditions of growth and starvation. *Arch. Microbiol.* 159 372–379. 10.1007/bf00290920

[B18] Li T. T. Tian W. L. Gu C. T. (2021). Elevation of *Lactococcus lactis* subsp. *cremoris* to the species level as *Lactococcus cremoris* sp. nov. and transfer of *Lactococcus lactis* subsp. *tructae* to *Lactococcus cremoris* as *Lactococcus cremoris* subsp. *tructae* comb. nov. *Int. J. Syst. Evol. Microbiol.* 71. 10.1099/ijsem.0.004727 33650946

[B19] Mateus C. Avery S. (2000). Destabilized green fluorescent protein for monitoring dynamic changes in yeast gene expression with flow cytometry. *Yeast* 16 1313–1323. 10.1002/1097-0061(200010)16:14<1313::AID-YEA626>3.0.CO;2-O 11015728

[B20] Nannen N. L. Hutkins R. W. (1991). Proton-translocating adenosine triphosphatase activity in lactic acid Bacterial1. *J. Dairy Sci.* 74 747–751. 10.3168/jds.s0022-0302(91)78220-9

[B21] Nugroho A. D. W. Kleerebezem M. Bachmann H. (2020). A novel method for long-term analysis of lactic acid and ammonium production in non-growing *Lactococcus lactis* reveals pre-culture and strain dependence. *Front. Bioeng. Biotechnol.* 8:580090. 10.3389/fbioe.2020.580090 33163481 PMC7580867

[B22] Ormö M. Cubitt A. B. Kallio K. Gross L. A. Tsien R. Y. Remington S. J. (1996). Crystal structure of the Aequorea victoria green fluorescent protein. *Science* 273 1392–1395. 8703075 10.1126/science.273.5280.1392

[B23] Otto R. Vije J. Brink B. T. Klont B. Konings W. N. (1985). Energy metabolism in *Streptococcus cremoris* during lactose starvation. *Arch. Microbiol.* 141 348–352. 10.1007/bf00428848

[B24] Papadimitriou K. Alegría Á. Bron P. A. de Angelis M. Gobbetti M. Kleerebezem M. (2016). Stress physiology of lactic acid bacteria. *Microbiol. Mol. Biol. Rev.* 80 837–890. 27466284 10.1128/MMBR.00076-15 PMC4981675

[B25] Pereira V. D. M. Neto D. C. Junqueira D. O. Karp S. Letti L. Magalhaes A. (2020). A review of selection criteria for starter culture development in the food fermentation industry. *Food Rev. Int.* 36 135–167.

[B26] Poolman B. Smid E. J. Veldkamp H. Konings W. N. (1987). Bioenergetic consequences of lactose starvation for continuously cultured *Streptococcus cremoris*. *J. Bacteriol.* 169 1460–1468. 10.1128/jb.169.4.1460-1468.1987 3558320 PMC211968

[B27] Price C. E. Santos F. B. D. Hesseling A. Uusitalo J. J. Bachmann H. Benavente V. (2019). Adaption to glucose limitation is modulated by the pleotropic regulator CcpA, independent of selection pressure strength. *BMC Evol. Biol.* 19:15. 10.1186/s12862-018-1331-x 30630406 PMC6327505

[B28] Rallu F. Gruss A. Ehrlich S. D. Maguin E. (2000). Acid- and multistress-resistant mutants of *Lactococcus lactis*: identification of intracellular stress signals. *Mol. Microbiol.* 35 517–528. 10.1046/j.1365-2958.2000.01711.x 10672175

[B29] Redon E. Loubiere P. Cocaign-Bousquet M. (2005). Transcriptome analysis of the progressive adaptation of *Lactococcus lactis* to carbon starvation. *J. Bacteriol.* 187 3589–3592. 10.1128/JB.187.10.3589-3592.2005 15866950 PMC1111995

[B30] Stuart M. R. Chou L. S. Weimer B. C. (1999). Influence of carbohydrate starvation and arginine on Culturability and amino acid utilization of *Lactococcus lactis* subsp. *lactis*. *Appl Environ Microbiol.* 65 665–673. 10.1128/AEM.65.2.665-673.1999 9925598 PMC91077

[B31] Šušković J. Kos B. Beganović J. Pavunc A. L. Habjanič K. Matošić S. (2010). Antimicrobial Activity – The most important property of probiotic and starter lactic acid bacteria. *Food Technol. Biotechnol.* 48 296–307.

[B32] Thomas T. D. Batt R. D. (1969). Synthesis of protein and ribonucleic acid by starved Streptococcus lactis in relation to survival. *J. Gen. Microbiol.* 58 363–369. 10.1099/00221287-58-3-363 5365928

[B33] van de Guchte M. Serror P. Chervaux C. Smokvina T. Ehrlich S. D. Maguin E. (2002). Stress responses in lactic acid bacteria. *Antonie Van Leeuwenhoek* 82 187–216. 12369188

[B34] van Tatenhove-Pel R. J. Zwering E. Solopova A. Kuipers O. P. Bachmann H. (2019). Ampicillin-treated *Lactococcus lactis* MG1363 populations contain persisters as well as viable but non-culturable cells. *Sci. Rep.* 9:9867. 31285492 10.1038/s41598-019-46344-z PMC6614399

[B35] Wegmann U. O’Connell-Motherway M. Zomer A. Buist G. Shearman C. Canchaya C. (2007). Complete genome sequence of the prototype lactic acid bacterium *Lactococcus lactis* subsp. *cremoris* MG1363. *J. Bacteriol.* 189 3256–3270. 10.1128/JB.01768-06 17307855 PMC1855848

[B36] Wels M. Siezen R. van Hijum S. Kelly W. J. Bachmann H. (2019). Comparative genome analysis of *Lactococcus lactis* indicates niche adaptation and resolves genotype/phenotype disparity. *Front. Microbiol.* 10:4. 10.3389/fmicb.2019.00004 30766512 PMC6365430

[B37] Zhang H. Lyu Z. Fan Y. Evans C. R. Barber K. W. Banerjee K. (2020). Metabolic stress promotes stop-codon readthrough and phenotypic heterogeneity. *Proc. Natl. Acad. Sci. U.S.A.* 117 22167–22172. 10.1073/pnas.2013543117 32839318 PMC7486758

