# Supplementary material for: Stationary Lactococcus cremoris: Energetic State, Protein Synthesis Without Nitrogen and Their Effect on Survival
Source: Front Microbiol. 2021 Dec 17;12:794316. doi: 10.3389/fmicb.2021.794316 (PMC8719527; doi:10.3389/fmicb.2021.794316)
Supplement: Supplementary file 1 [file Data_Sheet_1.pdf]

frontiers in Microbiology

Supplementary Information

Stationary *Lactococcus cremoris*: Energetic state, protein synthesis without nitrogen and their effect on survival

Authors

[1,2]Sieze Douwenga[†], [2,3]Rinke J. van Tatenhove-Pel[†], [2]Emile Zwering, [1,2,4]Herwig Bachmann[*]

[†] These authors have contributed equally to this work and share first authorship.

[1] TiFN. Agro Business Park 82, 6708 PW Wageningen, The Netherlands

[2] Systems Biology Lab, Amsterdam Institute for Molecules, Medicines and Systems, VU University Amsterdam, de Boelelaan 1108, 1081HV Amsterdam, The Netherlands

[3] Department of Biotechnology, Delft University of Technology, Van der Maasweg 9, 2629HZ, Delft, The Netherlands

[4] NIZO, Kernhemseweg 2, 6718ZB Ede, The Netherlands

* Correspondence: Herwig.Bachmann@nizo.com

Section 1. Batch growth of *L. cremoris*

L. cremoris cultures stop growing when they are carbon- or acid-limited

We cultured *L. cremoris* MG1363 in CDM with increasing glucose concentrations and analysed the final OD_{600}. We also measured the final pH, as *L. cremoris* produces lactic acid as fermentation-product. Figure 2A and Figure 2B show two regimes. In the first regime the final OD_{600} increases with increasing glucose concentration. At the same time the final pH of the culture decreases with increasing glucose concentration. In this regime the cultures enter stationary phase due to glucose starvation. In the second regime the final OD_{600} is not increasing anymore, and the pH stabilizes at a low level. In this regime glucose is still present in the cultures, but the cells entered stationary phase due to weak-acid uncoupling.

Increasing the buffer capacity increases the final pH of an acid-limited culture

Weak-acid uncoupling is driven by the extracellular protonated lactic acid concentration, which depends on the total amount of protonated and deprotonated lactic acid present, and on the extracellular pH (Figure 1). This means that increasing the buffer capacity of a medium will increase the pH at which *L. cremoris* becomes acid-limited: at a higher buffer capacity the same amount of lactic acid produced results in a smaller drop in pH, so at a given pH the protonated lactic acid concentration will be higher, and the critical protonated lactic acid concentration will be reached at a higher pH.

This effect is illustrated in Figure S1, which shows that at higher buffer capacities *L. cremoris* can consume more glucose and reach higher cell concentrations, while at the same time cultures become acid-limited at a higher pH.

Overall, these results show that increasing the buffer capacity increases the final pH of acid-limited *L. cremoris* cultures.

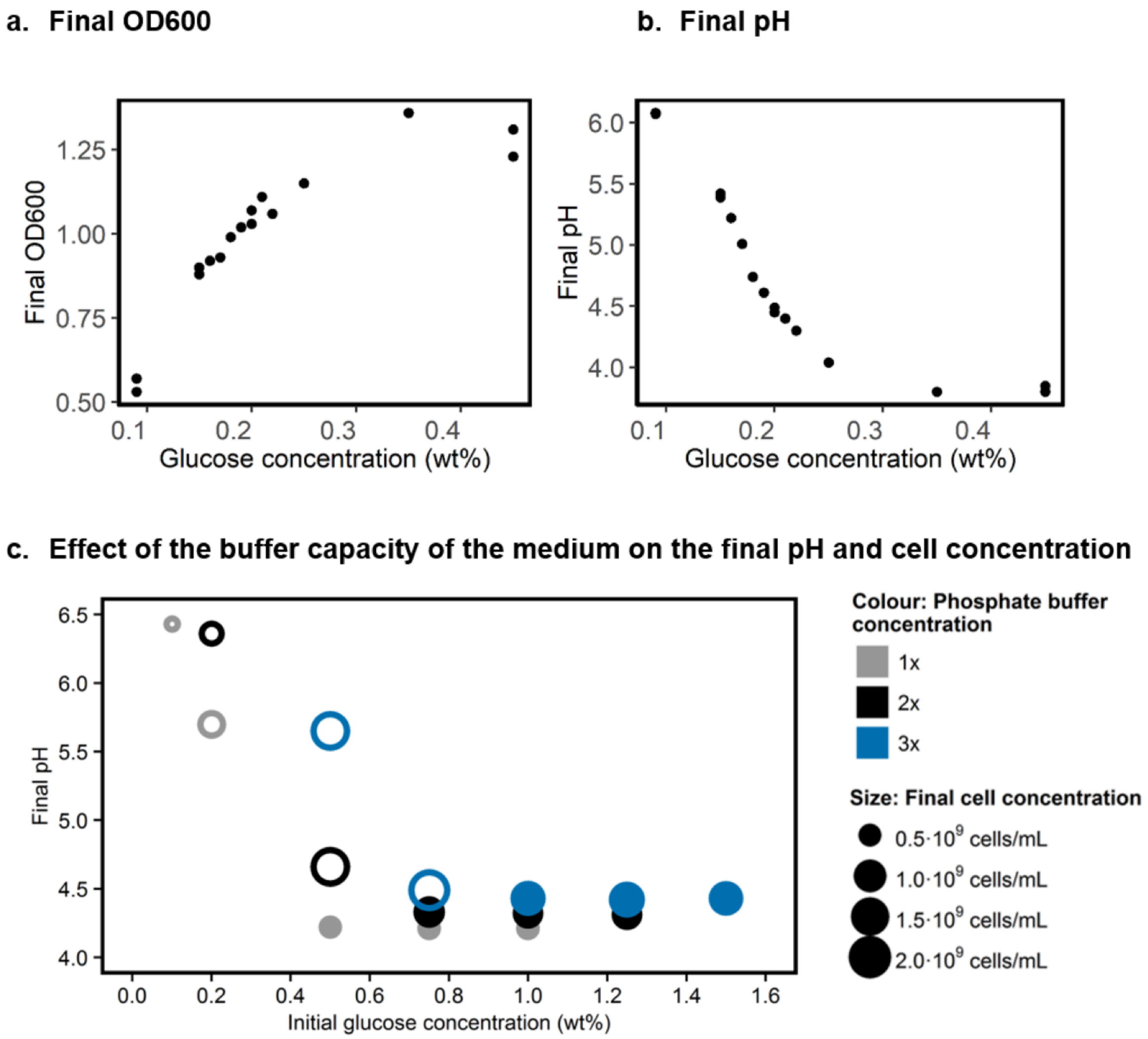

Figure S1. Final OD600 (a) and pH (b) of *L. cremoris* cultures. *L. cremoris* MG1363 was grown in CDM at different initial glucose concentrations. Final OD600 values (A) and final pH values (B) were measured. **(c) Effect of changing the phosphate buffer concentration in medium** *L. cremoris* MG1363 was grown in a different chemically defined medium, CDMtlf, a medium described by Otto et al.[1] with the following changes: 7.5 g/L K2HPO4, 9 g/L KH2PO4, 0.6 g/L NH4-citrate, 2.5 mg/L biotin, 0.02 mg/L riboflavin and no folic acid. Different phosphate buffer concentrations (fold-change compared to default phosphate buffer concentration) and different initial glucose concentrations were

used. After overnight growth we measured the final pH and cell concentration. We also tested whether glucose was present or absent: open symbols indicate that glucose was finished, closed symbols indicate that glucose was still present.

Section 2. Calibration curve to couple the fluorescence of *L. cremoris* MG1363_GFP to its intracellular pH

In previous work, the intracellular and extracellular pH of *L. cremoris* MG1363_GFP have been equilibrated using the membrane-uncouplers valinomycin and nigericin, and the corresponding fluorescence of the cells was measured[2]. We here used this data to make a calibration curve that couples the fluorescence signal of *L. cremoris* MG1363_GFP to its intracellular pH.

We first normalised the fluorescence of the cells to their fluorescence signal at pH 7.0, which is close to the optimal intracellular pH of *L. cremoris*[3].

We subsequently fitted a sigmoidal curve through the data:

$$A^- = \frac{1}{1 + \left(\frac{Ka}{pH}\right)^n}$$

In this equation A^- is the average fluorescence, as only the deprotonated form of GFP is fluorescent, and the pH is the intracellular pH, which equals the extracellular pH in these cells[2]. We fitted this equation to the data with Ka and *n* as variables (Figure S2), which resulted in Ka = 5.88±0.01 and n = 17.4±0.4.

Figure S2 shows that this calibration curve can be used to estimate the intracellular pH when it is between 4.5 and 7.0.

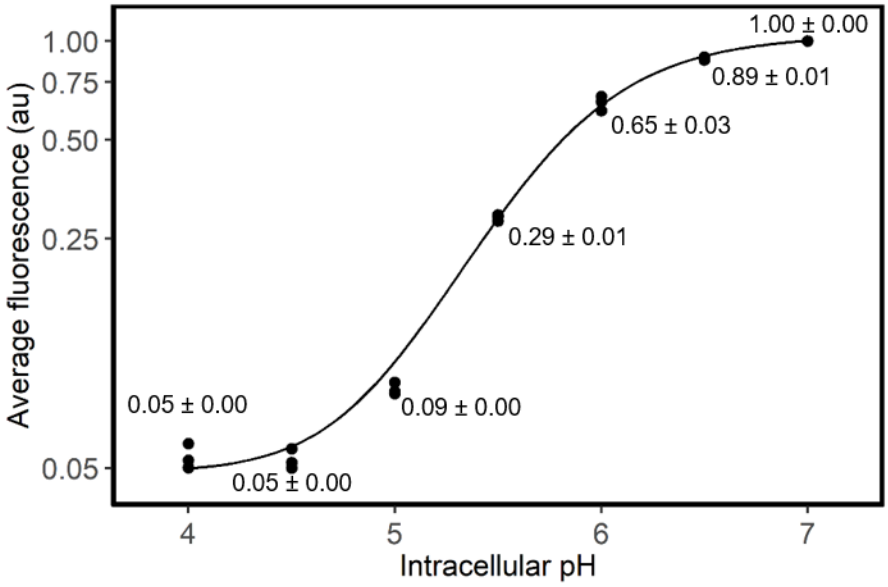

Figure S2. Calibration curve to couple the average fluorescence to the intracellular pH. pH values and average fluorescence signals for membrane-uncoupled cells were retrieved from previous data[2], and a Hill-equation was fitted through the data as described in section 2 of the supplementary information; n=3.

Section 3. Supplementary figures and tables.

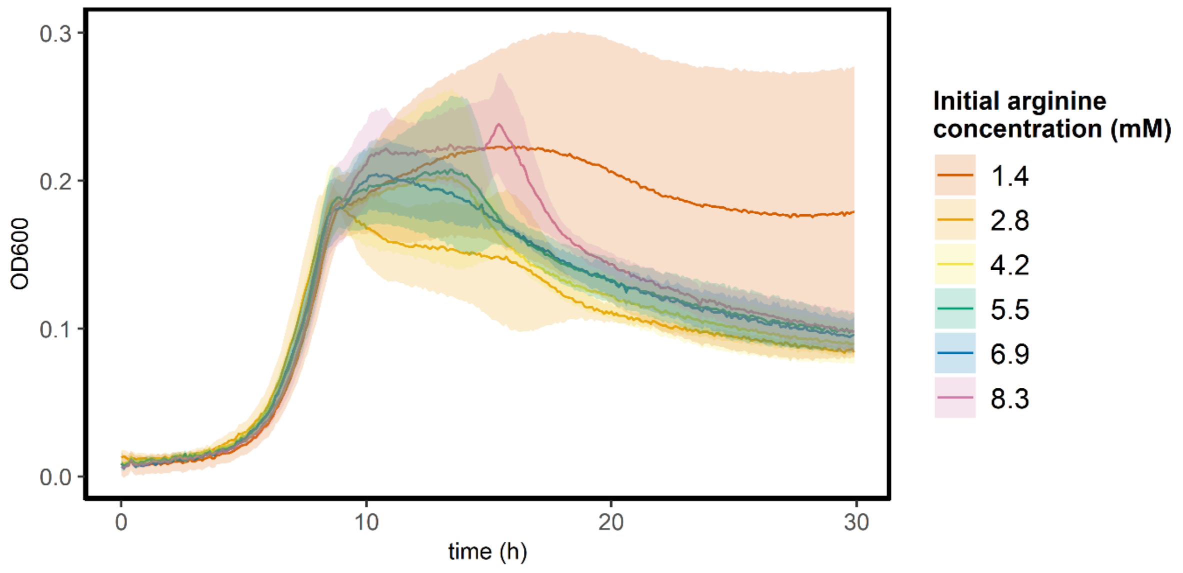

Figure S3. OD600 of growing and glucose starved stationary *L. cremoris* cultures. *L. cremoris* was grown in medium with six initial arginine concentrations, and the carboxyfluorescein (Figure 3) OD_{600} (this figure) were followed in time. Lines show the mean, and shades the standard deviation (n = 28 for 1.4 mM arginine, n = 4 for all other arginine concentrations). The data indicates that at all arginine supplementation levels cells go into stationary phase after approximately 9 hours. The observed fluctuations in OD600 after 9 hours, are most likely caused by cell sedimentation, but this has no major influence on determining the time at which cells go into stationary phase.

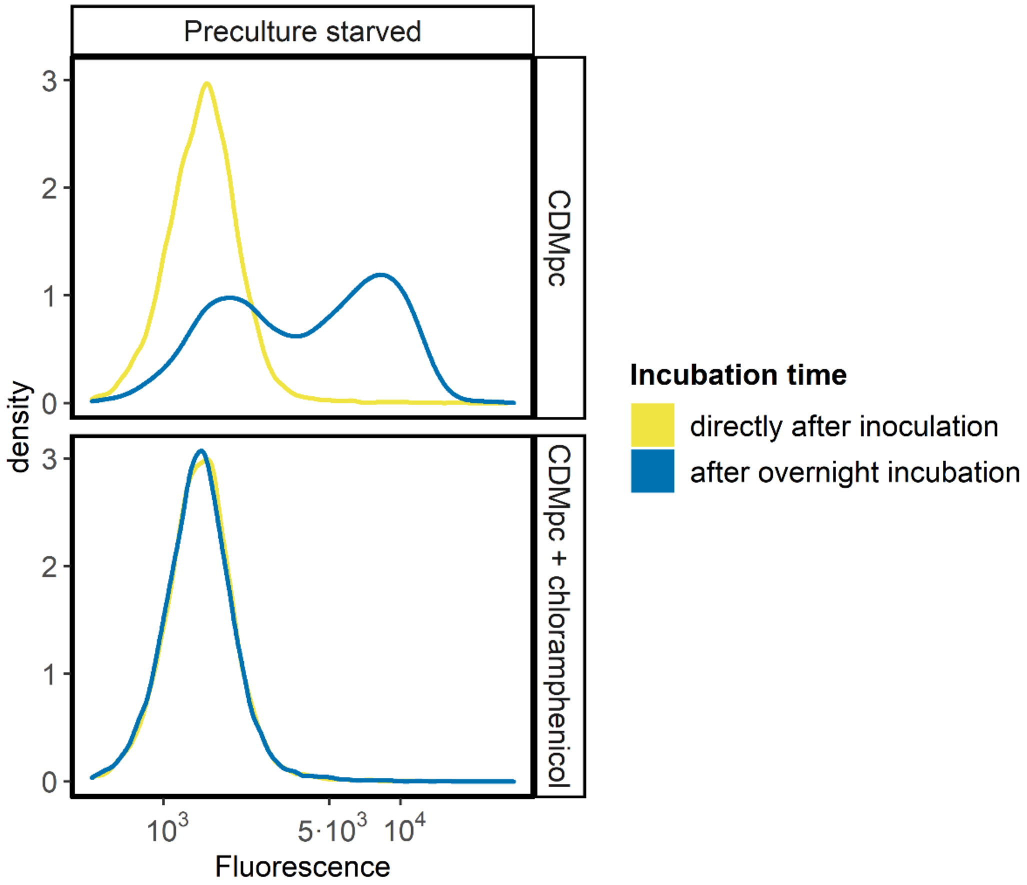

Figure S4. Fluorescence of stationary *L. cremoris* cells in CDM with no available carbon source. Glucose starved stationary pre-cultures of *L. cremoris* MG1363_GFP were diluted in CDM without an available carbon source, and CDM without an available carbon source supplemented with 20 mg/L chloramphenicol. Their fluorescence was measured directly after inoculation and after overnight incubation.

**Medium**	**pH**	**Average fluorescence after 50 minutes**
PBS + 20 mM lactate	4.7	622
PBS + 20 mM lactate + 0.5 w/v% glucose	4.7	2518
PBS + 20 mM lactate + 2 mM arginine	4.7	2517

Table S1. Average fluorescence of cells in PBS of pH 4.7 with 20 mM lactate. Glucose starved stationary *L. cremoris* MG1363_GFP cells were washed and transferred to PBS of pH 4.7 supplemented with 20 mM lactate. Glucose or arginine were added and the average fluorescence of cells was determined using flow cytometry after 50 minutes, to analyse whether cells could maintain a pH gradient in these conditions.

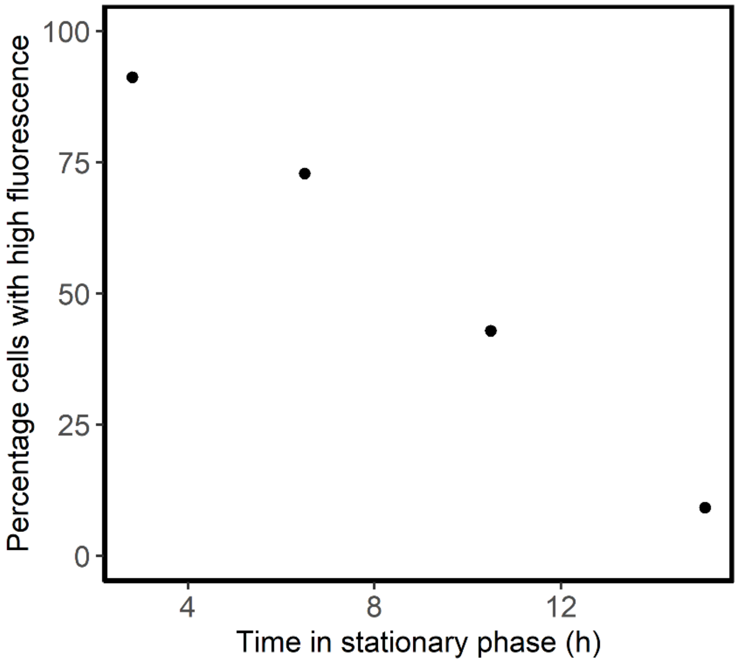

Figure S5. Percentage of cells with a high fluorescence at various stationary phase lengths. In Figure 6b the fluorescence increase of *L. cremoris* at various stationary phase lengths is shown. Figure 6b showed two subpopulations for each stationary phase length, one with a high fluorescence and one with a low fluorescence. In this figure we show the percentage of cells with a high fluorescence vs. their time in stationary phase.

References

1 Otto, R., Vije, J., Brink, B. t., Klont, B. & Konings, W. N. Energy metabolism in Streptococcus cremoris during lactose starvation. *Archives of Microbiology* **141**, 348-352, doi:doi:10.1007/BF00428848 (1985).

2 van Tatenhove-Pel, R. J., Zwering, E., Solopova, A., Kuipers, O. P. & Bachmann, H. Ampicillin-treated Lactococcus lactis MG1363 populations contain persisters as well as viable but non-culturable cells. *Scientific reports* **9**, 9867, doi:10.1038/s41598-019-46344-z (2019).

3 Even, S., Lindley, N. D., Loubière, P. & Cocaign-Bousquet, M. Dynamic response of catabolic pathways to autoacidification in Lactococcus lactis: transcript profiling and stability in relation to metabolic and energetic constraints. *Molecular microbiology* **45**, 1143-1152, doi:10.1046/j.1365-2958.2002.03086.x (2002).
